# Current Status and Perspectives on the Application of CRISPR/Cas9 Gene-Editing System to Develop a Low-Gluten, Non-Transgenic Wheat Variety

**DOI:** 10.3390/foods10102351

**Published:** 2021-10-02

**Authors:** Anil K. Verma, Sayanti Mandal, Aadhya Tiwari, Chiara Monachesi, Giulia N. Catassi, Akash Srivastava, Simona Gatti, Elena Lionetti, Carlo Catassi

**Affiliations:** 1 Celiac Disease Research Laboratory, Polytechnic University of Marche, 60123 Ancona, Italy; c.monachesi@pm.univpm.it; 2 Institute of Bioinformatics and Biotechnology, Savitribai Phule Pune University, Ganeshkhind Road, Pune 411007, Maharashtra, India; mandalsayanti@gmail.com; 3 Department of System Biology, MD Anderson Cancer Center, Houston, TX 77030, USA; ATiwari3@mdanderson.org; 4 Laboratory of Cell Biology, Department of Orthopaedic Surgery, University Hospital of Tübingen, Waldhörnlestraße 22, D-72072 Tübingen, Germany; 5 Division of Pediatrics, DISCO Department, Polytechnic University of Marche, 60123 Ancona, Italy; giulia.catassi@gmail.com (G.N.C.); simona.gatti@hotmail.it (S.G.); m.e.lionetti@univpm.it (E.L.); c.catassi@univpm.it (C.C.); 6 Department of Molecular Biology, Cell Biology and Biochemistry, Brown University, Providence, RI 02906, USA; akashsriv1@gmail.com; 7 Mucosal Immunology and Biology Research Center, Division of Pediatric Gastroenterology and Nutrition, Massachusetts General Hospital, Boston, MA 02114, USA

**Keywords:** celiac disease, CRISPR/Cas9, RNAi, α-gliadin, low-gluten, non-transgenic wheat

## Abstract

Wheat gluten contains epitopes that trigger celiac disease (CD). A life-long strict gluten-free diet is the only treatment accepted for CD. However, very low-gluten wheat may provide an alternative treatment to CD. Conventional plant breeding methods have not been sufficient to produce celiac-safe wheat. RNA interference technology, to some extent, has succeeded in the development of safer wheat varieties. However, these varieties have multiple challenges in terms of their implementation. Clustered Regularly Interspaced Short Palindromic Repeats-associated nuclease 9 (CRISPR/Cas9) is a versatile gene-editing tool that has the ability to edit immunogenic gluten genes. So far, only a few studies have applied CRISPR/Cas9 to modify the wheat genome. In this article, we reviewed the published literature that applied CRISPR/Cas9 in wheat genome editing to investigate the current status of the CRISPR/Cas9 system to produce a low-immunogenic wheat variety. We found that in recent years, the CRISPR/Cas9 system has been continuously improved to edit the complex hexaploid wheat genome. Although some reduced immunogenic wheat varieties have been reported, CRISPR/Cas9 has still not been fully explored in terms of editing the wheat genome. We conclude that further studies are required to apply the CRISPR/Cas9 gene-editing system efficiently for the development of a celiac-safe wheat variety and to establish it as a “tool to celiac safe wheat”.

## 1. Introduction

Common wheat (*Triticum aestivum*, 2*n*  =  6*x*  =  42, AABBDD) is a preferred staple food worldwide [1]. During 2018/19, the total global wheat consumption was 734.7 million metric tons, which increased by 759 million metric tons during 2021 [2]. However, in a huge number of individuals, the consumption of gluten (a storage protein of wheat) triggers several gluten-related disorders (GRDs), including celiac disease (CD), which affects 1–2% of the world population [3]. CD is a T-cell mediated chronic enteropathy caused by the ingestion of immuno-dominant gluten peptides in genetically predisposed individuals who possess a specific human leukocyte antigen (HLA)-DQ2 and/or -DQ8 alleles [4,5,6]. Following a life-long strict gluten-free diet (GFD) is the only accepted treatment for CD [7]. Adherence to a strict GFD shows absolute regression in the celiac-associated symptoms (diarrhea, anemia, failure to thrive, weight loss, etc.) and is also suggested for other GRDs [4,5,7,8]. Gluten is a ubiquitous protein that is used universally not only in cereal-based products but also in numerous food and non-food industries [9,10]. Therefore, complete elimination of gluten from the diet is difficult [9,10]. Following a strict GFD also compromises the quality of life (QOL) of CD patients [11].

Gluten protein is primarily comprised of two classes of proteins, i.e., gliadins and glutenins. While gliadin makes dough viscous, glutenins provide a fine baking quality to wheat [12]. The existence of gliadins and glutenins as well as the balance of these two forms of proteins is critical for flour quality. Gliadin is further sub-divided into α-, γ-, and ω-subfractions, out of which α-gliadin primarily contains the critical epitopes that are responsible for CD development [13]. There are two fractions of glutenins, i.e., low and high molecular weight glutenins [12]. Gliadin is encoded by multiple gene families that are arrayed at *Gli-2* loci on chromosome 6A, B, and D on specific loci in a repetitive sequence fashion [4,13,14,15,16]. α-gliadin contains a 33-mer peptide that is particularly rich in proline-glutamine sequences, and some of these α-gliadins are responsible for the development of CD. Human intestinal and pancreatic enzymes are unable to completely digest the complex amino acid sequence of α-gliadin, that is broken down into relatively larger peptides [4,17]. These peptides pass through intercellular junctions and enter in the lamina propria, where the tissue transglutaminase enzyme deamidates this gluten fraction. This modified fraction is recognized by the HLA-DQ heterodimers that are attached to antigen presentation cells. The HLA–gluten complex triggers T-cells to induce a pro-inflammatory cascade, which eventually leads to CD [17].

Wheat was introduced into the human diet about 10,000–12,000 years ago [18]. The first domesticated wheat varieties were diploid and tetraploid. Einkorn wheat only had one genome, i.e., the A genome (diploid). This wheat variety was designated as *T. monococcum* and is rarely consumed by humans nowadays [18]. Tetraploid wheat was domesticated simultaneously with diploid wheat and contains two genomes (AA and BB); hence, it was termed tetraploid wheat. Durum wheat (*T. durum* or *T. turgidum*) is a tetraploid species of wheat that is mostly used to prepare pasta [18].

The currently most used bread wheat/common wheat (*T. aestivum*) is an allohexaploid species with three genomes (AA, BB, DD) resulting from natural hybridization between a tetraploid *T. turgidum* (*dicoccum*) carrying the AA, BB-genome, and the wild diploid species *T. tauschii* (DD-genome) [19]. While the introduction of the D-genome improved the bread-making properties of wheat, most of the immunogenic peptides in CD are encoded by the D-genome [18]. α-gliadin, which is encoded on D-genome, is more immunogenic and more easily recognized by the intestinal T-cells. Preliminary shreds of evidence suggest that primitive wheat (diploid or tetraploid) was safer and less immunogenic compared to currently used hexaploid wheat, as ancient wheat varieties had less immune-dominant protein fractions. However, this is strictly dependent on the particular genotype, not on the species [18,20]. Wheat varieties with low T-cell stimulatory epitopes may reduce the chances of developing CD. Exposure to an improved wheat variety with low-immunogenic wheat may not cause an intense immunological trigger to CD patients; hence. it could be useful for CD management [12,21].

Numerous efforts have been executed to develop a wheat variety with a lower percentage of immunological peptides (α-, ω-, and γ-gliadin), primarily by applying a combination of conventional mutation and breeding methods and RNA interference (RNAi) technology. However, a low-immunogenic wheat variety has not been able to be developed so far. [12,21,22,23,24].

In recent years, gene-editing techniques such as zinc finger nuclease (ZFN), and transcription activator-like effector nucleases (TALEN) have emerged as a promising approach to edit or delete the gluten fractions in wheat [25]. Another promising gene-editing tool, i.e., clustered regularly interspaced short palindromic repeats-associated nuclease 9 (CRISPR/Cas9) has evolved as a popular and novel second-generation genome-editing tool in science, medicine, and biotechnology. The CRISPR/Cas9 gene-editing system can remove or reduce the toxic fractions of gluten, resulting in a gluten-free or low-gluten wheat [13]. This gluten-free or low-gluten wheat would be a healthier choice for CD and GRD patients [12]. The use of hypoimmunogenic wheat flour in the preparation of gluten-free food or gluten-free products may also be useful for reducing the increasing burden of gluten cross-contamination [26]. Due to genetic redundancy and genome complexity, wheat biology has straggled behind in adopting CRISPR/Cas9-based genome modifications. The key challenge now is to fully exploit the genome-editing ability of CRISPR/Cas9 to precisely alter gliadin genes, suppressing their immunogenic capability while maintaining their functionality and organoleptic properties.

So far, only a few studies have reported the application of CRISPR techniques to produce low-immunogenic/gluten-free wheat with novel agronomical traits. To the best of our knowledge, this review is among the first reports to provide an outline of the current status and contribution of CRISPR/Cas9 applications in the editing of the wheat genome. This article will help in bridging the research gaps that currently exist towards the development of wheat lines devoid of immunogenic gluten.

## 2. Literature Review

From January to April 2021, published literature related to the application of CRISPR to develop a low-immunogenic wheat variety was searched using the keywords <celiac and CRISPR>, <CRISPR in celiac disease>, and <Wheat engineering with CRISPR/Cas9>, <Low-immunogenic wheat and CRISPR> on electronic databases such as PubMed, Google Scholar, CrossRef, and CiteFactor. We also searched the references from the published articles that were found. No publication date was imposed. Only original articles published in the English language applying CRISPR/Cas9 for gene-editing in wheat crops were included. Review articles, protocols, scientific presentations, and Ph.D. theses were excluded; however, such articles were only used for reading purposes. Following these criteria, 68 studies were explored in total. Of them, 23 articles were found to be appropriate for the topic.

## 3. Genome-Editing Techniques: Tools That Alter the Genetic Code

Genome editing or gene editing is an advanced technique that permits researchers to perform specific alterations in the genome of living cells. During 1970s, the development of genetic engineering (manipulation of DNA or RNA) opened up innovative possibilities in genome editing [27].

The main concept behind genome-editing techniques is to employ engineered endonucleases to create a site-specific DNA double-strand break (DSB), which is repaired either by non-homologous end joining (NHEJ) or by homologous recombination (HR) [12,25,28,29]. Genome-editing techniques have been categorized into two generations: (1) first-generation (i.e., mega-nucleases, ZFNs, TALEN) and (2) second-generation (e.g., CRISPR) gene-editing tools [25,30]. CRISPR is the latest gene-editing tool and is highly accurate, rapid, simple, and comparatively cheaper than other gene-editing tools [31,32]. The CRISPR/Cas9 system has been successfully applied for plant genome (Arabidopsis, rice, maize, and tomato) improvement and in various human diseases such as gastrointestinal, hematologic, viral, and cancer [13,33]. In a recent study, CRISPR/Cas9 significantly inhibited tumor cell growth as well as the migration and invasion of breast cancer cells [34].

## 4. CRISPR/Cas9: A New Era of Genome Editing

The concept of CRISPR/Cas9 has been adopted from the defense machinery of bacteria [25,32,35]. When a virus (bacteriophage) attacks bacteria, the bacteria capture snippets of the genetic material of the virus and synthesizes DNA segments known as CRISPR arrays [25,32,35]. These CRISPR arrays memorize the virus, and on future invasions of the same or similar viruses, the bacteria then synthesize the RNA segments from the CRISPR arrays that target that virus. Bacteria use the Cas9 enzyme to cleave the targeted viral DNA sequence that eventually neutralizes the virus [36].

The CRISPR genome-editing system requires the design of guide RNA (gRNA) 20 nucleotides, which is complementary to the DNA stretch within the target gene. Along with the gRNA, the system also requires the Cas9 endonuclease, which together forms a ribonucleoprotein (RNP) complex that creates DSB in complementary DNA sequences [36,37]. In various human diseases, including neurodegenerative conditions, acquired immunodeficiency syndrome, and β-thalassemia, the CRISPR/Cas9 mechanism has been implemented effectively [13,33]. Recently, CRISPR/Cas9 has become a promising technique for trait improvement or functional genomics studies in various commercially relevant crops (*Oryza sativa*, *Zea mays*, *Solanum lycopersicum*, *S. tuberosum*, *Hordeum vulgare,* and *T. aestivum*). The use of the CRISPR/Cas9 system in plant genetic engineering is a relatively more contemporary and widely adopted tool for genome editing than ZFNs and TALENs [38,39]. The simplicity, multiplexed mutations, and robustness of CRISPR/Cas9 make it a preferred choice over first-generation genome-editing tools [40].

## 5. CRISPR/Cas9: The Machinery

The CRISPR/Cas9 system is present in diverse living organisms and fundamentally has a comparable core genetic organization [41,42]. They generally have multiple Cas genes encoding the Cas protein and several repeat DNA elements interspersed with short “spacer” sequences derived from foreign DNA. The AT-rich spacer sequence constitutes a code for the respective foreign genetic element that is used by the host prokaryotic to quickly identify any homologous sequence subsequently entering the host cells [43].

**Figure 1:**
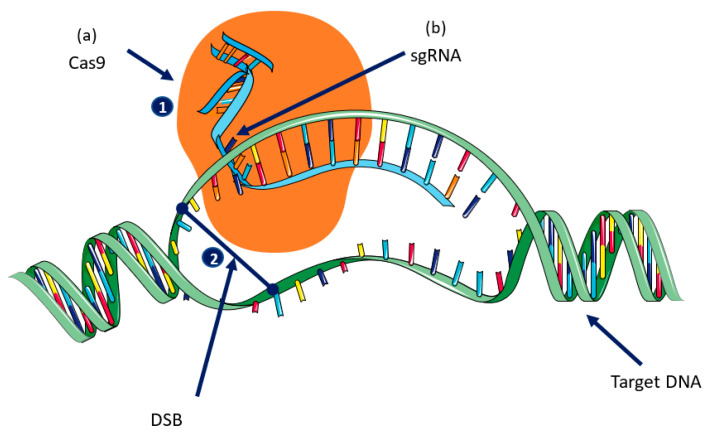
Mechanism of CRISPR/Cas9-mediated gene editing: there are two key components of CRISPR/Cas9: (**a**) Cas9 and (**b**) single guide RNA (sgRNA) **1:** The Cas9 nuclease is guided to its target DNA by the sgRNA. **2:** Cas9 causes a double-strand break (DSB) in the DNA that is repaired using either a non-homologus joining (NHEJ) or by homologus recombination (HR).

There are two main components of CRISPR: (1) single guide RNA (sgRNA), which is complementary to the target sequence, and (2) the Cas9 gene, which is adapted from *Streptococcus pyogenes* (SpCas9) and requires a G-rich (5′-NGG-3′) PAM (protospacer-adjacent motif) site that is responsible for generating DSB at a predesigned target DNA site [32,37,44]. sgRNA is a small sequence of nucleotides (18–21 nucleotides) that is complementary to the target DNA, and that has three PAM sites at the 3′ end followed by an RNA scaffold [45]. The Cas9 protein comprises two functional domains: (1) the large recognition (REC) domain, which is the largest domain and is responsible for gRNA binding, and the (2) RuvC domain, which is a nuclease domain that cuts the single-stranded DNA. The NUC domain has two conserved endonuclease sites (RuvC and HNH) and a PAM interacting site. RuvC cleaves the non-complementary strand while HNH cleaves the complementary sequence of the sgRNA [12,36,45] (Figure 1).

To neutralize foreign DNA in bacterial cells, the CRISPR/Cas9 system works in three stages [46,47]:

**Stage I, acquisition stage:** The invading DNA is recognized, and the spacer sequence is obtained from the target DNA. The repeated DNA sequence is inserted into the host CRISPR array to build an immunological memory [48,49].
**Stage II, expression stage:** The Cas9 protein is expressed at this stage, and the CRISPR array is transcribed into a precursor RNA transcript (pre-crRNA). The pre-crRNA and Cas9 protein are then hybridized by a non-coding trans-activating CRISPR-RNA (crRNA) and are processed into a mature RNA unit known as crRNA [50,51].
**Stage III, interference stage:** In the final stage, the mature crRNA directs the Cas9 protein to identify the DNA of interest, resulting in the cleavage and degradation of the invading foreign DNA [52,53].

The Cas9 endonuclease cleaves the DNA to generate blunt-ended DSB in the host genome, triggering a cellular DNA repair mechanism. The host DNA repair mechanism may either follow an NHEJ with small random insertion/deletion or by HDR, thus resulting in genome editing at the target locus [54]. In NHEJ, a highly error-prone repair mechanism, DSB, joins back together with the endogenous repair machinery, which generally introduces random insertions and deletions of the DNA. This could potentially lead to the disruption of the codon-reading frame and often results in gene knockout by forming a frameshift and premature stop codon. Alternatively, if a donor DNA template homologous to the sequence surrounding the DSB site remains available, the error-free HDR pathway is initiated, whereby precise deletions or insertions of the coding sequences can be achieved, leading to gene knock-in or deletion. The NHEJ leads to ablation gene mutation and can be used to generate a loss of function effect, whereas HDR can introduce precise changes in the genome by adding specific point mutations or by varying the length of the DNA segments [44,45].

## 6. CRISPR/Cas9: Challenges and Consequences in the Wheat Genome

The CRISPR/Cas9 system is a dominant gene-editing tool that has been successfully applied in more than 20 agronomically important crops species so far, and its application has led to yield improvements, disease resistance, biotic and abiotic stress, etc. [55]. In recent years, the CRISPR/Cas9 system has been employed in model plants such as *Arabidopsis thaliana* and *Nicotiana benthamiana*. Subsequently, this genome editing has been employed in major crops such as rice, wheat, maize, oilseeds, tomato, soybean, cotton, and potato [56]. Even though the CRISPR/Cas9 method has been validated in various crops, large-scale implementation in editing α-gliadins in wheat is still lacking. One of the major difficulties was the complex wheat genome. Hexaploid wheat *T. aestivum* (Bread Wheat) has a large genome (approximately 17 Gbp) and has a high content of the repetitive sequences. This robust sequence prevents the insertion of target mutants in the genome and makes the editing process difficult [12]. Apart from this, modern wheat is an allohexaploid, i.e., it is the result of a series of naturally occurring hybridization events among *T. urartu* (A genome donor), *T. speltoides* (B genome donor), and *T. tauschii* (D genome donor) [57,58]. Due to the large and complex three homologous copies of genes (A, B, and D) in the genome, targeting multiple copies of a gene has always been challenging for gene-editing techniques [12,25].

However, due to the orthologues of the Cas9 gene, CRISPR/cas9 is now capable of targeting multiple genes simultaneously [13,59]. Currently, the CRISPR/Cas9 system is being used in the development of a low-immunogenic wheat variety. [12,31,60].

## 7. Application of CRISPR/Cas9 System in Wheat Genome Editing

In 2014, for the first time, the CRISPR/Cas9 system was used successfully in wheat protoplasts to edit the *TaMLO* gene (Mildew resistance locus O) [61]. The CRISPR TaMLO knockout lines have been successfully established to increase resistance against *Blumeria graminis* f. sp. *Tritici* (Btg), the causal organism of powdery mildew disease. The seventy-two T_0_ lines obtained by biolistic particle transformation were analyzed for T7 endonuclease 1 (T7E1) restriction enzyme digestion, with four lines being reported to be edited for the T7E1 restriction enzyme site [62]. A T-DNA-based delivery system was commonly used to introduce sequence-specific nucleases (SSNs) and the gRNA. However, DNA–virus-based amplicons were used as an efficient construct delivery method and led to several-fold increases in terms of gene targeting efficiencies. The application of Geminivirus-based DNA replicons, such as a wheat dwarf virus (WDV) in wheat, resulted in a 12-fold increase in CRISPR/Cas9 expression compared to the ubiquitin reference gene, suggesting that it could be a future tool for genome engineering for complex genomes [63]. In another study, Kim et al., (2018) demonstrated gene editing in wheat protoplasts for *dehydration-responsive element-binding protein 2* (*TaDREB2*) and *ethylene-responsive factor 3* (*TaERF3*) using the wheat U6 snRNA promoter [60]. They successfully transfected nearly 70% of protoplasts and confirmed the expressions of these edited genes with the T7 endonuclease assay. The two major pitfalls of CRISPR-mediated gene editing in crops (CMGE) were transgene integration and off-targeting into the genome. Off-target mutations were more common in crops with higher ploidy levels as well as in genes with a large number of paralogs. This shortcoming was overcome by using a biolistic delivery method for the CRISPR/Cas9 ribonucleoproteins (RNPs). However, RNP-based biolistic delivery offers a transient expression of CRISPR/cas9, and it also reduces the chances of *off-target* effects [31]. Later, in 2017, Liang et al. demonstrated the use of CRISPR/Cas9 RNP complex genome editing for grain morphometric traits such as grain length (GL), width (GW) genes *TaGW2,* and *TaGASR7* in *T. aestivum*. This complex reduced off-target effects, as no off-targets were detected in the mutant *T. aestivum* population, and in addition, the complex became degraded in vivo. This DNA-free editing method had an advantage over traditional backcross breeding, which is a laborious and time-consuming procedure [64]. However, this method had some limitations, including low-efficiency rates compared to CRISPR/Cas9 DNA binary delivery systems. The RNP method is a more economical approach to achieve CRISPR/Cas9-based genome editing in perennial crop species if these limitations are overcome. Similarly, Wang W. et al., (2018) demonstrated the multiplexed gene editing of three wheat genes, *TaGW2* (a negative regulator of grain traits), *TaLpx-1* (lipoxygenase, which confers resistance to *Fusarium graminearum*), and *TaMLO* (loss of function, confers resistance to powdery mildew resistance), using the wheat U3 snRNA promoter [59]. Genome-editing efficiency was validated in wheat protoplasts, and the DNA was evaluated for mutations by next-generation sequencing (NGS) followed by *Agrobacterium*-mediated transformation and mutant screening. T_0_, T_1_, T_2_, and T_3_ were then subjected to statistical and phenotypic analysis, and three homeologous copies were observed for gene-editing efficiencies in wheat. In another study, the male sterility gene, i.e., *Ms1* (male sterility 1) was targeted by CRISPR/Cas9 vectors, resulting in the generation of complete sterility in commercial wheat cv. Fielder and Gladius [65]. In 2018, Sánchez-León et al. used particle bombardment to demonstrate the potential of CRISPR/Cas9, this time with two gRNAs delivered separately. They focused on genes that encode α-gliadins, seed storage proteins that have an epitope linked to CD. Twenty-one mutant lines in bread wheat and six in durum wheat were developed, both of which showed a significant reduction in α-gliadins and had up to 35 genes edited in a single line [12]. Howells et al. (2018) delivered gRNAs into wheat cells using *Agrobacterium tumefaciens*-mediated transformation, for example, to target the *TaPDS* gene, a gene that encodes phytoene desaturase [66]. Interestingly, Zhang et al. (2019) generated heritable targeted mutation in *TaPinb*, *TaDA1*, *TaDA2,* and *TaNCED1*. The combination of the *Agrobacterium*-mediated transformation process and the CRISPR/Cas9 gene-editing system greatly increased the mutagenesis efficiency in T_0_ generation. High editing frequency was observed in subsequent T_1_ and T_2_ generations. Since CRISPR/Cas9 activity is stable throughout generations, *Agrobacterium*-mediated transformation in wheat proved to be an ideal approach for genome editing [67]. 

Furthermore, *Agrobacterium*-mediated transformants contain only one or a few copies of the transgene, and transgene-free mutant lines are reasonably simple to acquire [68]. Kamiya et al. (2020) developed PCR-RFLP, a rapid method for detecting edited mutations in wheat that was validated by genomic clone sequencing. Three *TaNP1* homoeo-alleles, which encode a putative glucose-methanol-choline oxidoreductase and that are needed for male sterility, were edited using the optimized CRISPR/Cas9 method. It was also demonstrated that having only one wild-type copy of each of the three *TaNP1* genes was enough to maintain male fertility [69]. In a recent study, in order to reduce the expression of asparagine synthetase in grain without affecting its expression in any other part of the plant, Raffan et al. (2021) targeted the *TaASN2* gene in *T. aestivum* cv. Cadenza using the CRISPR/Cas9 system. The study provided strong evidence that very low-asparagine commercial wheat varieties can be produced, allowing for the development of lower-acrylamide bread, cereals, biscuits, and other wheat-based foods [70].

The abovementioned studies successfully demonstrate that the CRISPR/Cas9 system has emerged as an effective tool to enable precise genome manipulation for the development of new wheat cultivars with improved novel traits. These studies have documented how CRISPR/Cas9 has been successfully employed in the wheat genome to improve disease resistance, stress tolerance, increase yield, and nutritional improvement. We have summarized the twenty-three studies that used CRISPR/Cas9-mediated gene editing in wheat varieties in Table 1.

## 8. RNA Interference (RNAi): Biology

The discovery of RNA-induced gene silencing provided a feasible alternate gene analysis technique through the simultaneous knockdown of the expression of multiple related gene copies. RNAi or RNA-silencing was discovered in *Caenorhabditis elegans* and plants during the late1990s as a post-transcriptional gene silencing (PTGS) mechanism that is able to target specific messenger RNA (mRNA) sequences and to downregulates protein expression [29,81,82,83]. RNA interference involves four main stages: (1) double-stranded RNA cleavage by the Dicer, (2) silencing complex (RISC) development, (3) silencing complex activation, and (4) mRNA degradation.

The first step in RNAi is the transmission of dsRNA into the cell, which is completely homologous to the target gene in sequence. The Dicer enzyme recognizes dsRNA and converts it into double-stranded short interfering RNA (siRNA) nucleotides of varying lengths in an ATP-dependent reaction, depending on the species. In the second step, the siRNAs produced by Dicer are integrated into the RNA-induced silencing complex (RISC), a multicomponent nuclease complex whose ability to conduct RNAi is inactive in this form [29,84]. In an ATP-dependent process, a helicase unwinds the siRNA duplex and further remodels the complex to form an effective RISC in the third step. The final step is to recognize and cleave mRNA that is complementary to the siRNA strand present in RISC. The target mRNA is cleaved into 22 nucleotide-long fragments, resulting in gene suppression or in the alteration of gene expression [85]. When cleavage comes to an end, the RISC leaves, and the siRNA is ready to be used in another mRNA recognition and cleavage period [86,87].

## 9. Role of RNAi in Modifying the Wheat Genome

Wheat RNAi has been successfully used to target a wide range of genes to date, but it has also been used to down-regulate protein encoded by multigene families, such as gliadins and glutenins [88,89]. In a short communication published by Gil-Humanes et al. in 2008, the authors used RNA interference to suppress the expression of particular γ-gliadins, demonstrating the feasibility of systematically silencing specific groups of gluten proteins. There were seven transgenic lines, all of which displayed decreased γ-gliadin content. The seven transgenic plants were fully fertile, and the grain morphology and seed weight were comparable to the wild-type grain morphologies and seed weights. The proportion of γ-gliadins was decreased by about 55–80% in the BW208 lines and by about 33–43% in the BW2003 lines as a result of this silencing [84]. In another influential study published in 2010, Gil-Humanes et al. down-regulated the gliadin expression (up to 63–93% for α-gliadin and 35–81% for ω-gliadin) in bread wheat by designing a set of hpRNAs containing a fragment of 361 bp that is widely conserved among α-, ω-, and γ- gliadins. There was a 1.5–2 log reduction in the sum of the DQ2-α-II and DQ2-γ-VII epitopes and at least a 1 log reduction in the amount of DQ8-α-I and DQ8-γ-I epitopes in five of the transgenic lines. For three of the transgenic wheat lines, whole gluten extracts were unable to produce T-cell responses and had decreased responses for six transgenic lines [90]. Again in 2014, Gil-Humanes et al used flour from these transgenic wheat lines to develop a high-quality bread. The baking and sensory properties as well as the overall approval of the reduced-gliadin breads were comparable to those of regular flour but with up to 97% less gliadin content. Furthermore, low gliadin flour enhanced the nutritional properties because their lysine levels were considerably higher than that of regular wheat [91].

In a recent study, Haro et al. (2018) compared the digestibility of low-gliadin wheat (E82, low gliadin content, and reduced LMW glutenins) developed by the RNAi system from regular gluten-free bread in a subset of patients with no-celiac gluten sensitivity (NCGS). The findings indicated that eating low-gliadin E82 bread for one week was well accepted by NCGS patients, as the clinical effects were similar to those seen with gluten-free bread, and no variations in sensory parameters were observed. The data showed that the consumption of E82 bread does not cause adverse clinical symptoms, induces positive changes to the composition of the gut, increases butyrate-producing bacteria, and promotes the bacterial profile of the intestines, which plays a major role in gut permeability improvement in NCGS patients. However, this study did not address the relationship between the bacterial and fungal species of the gut microbiota. Further studies are needed to investigate bacterial and fungal microbiota modification in the gut upon the consumption of E82 bread [92].

These study findings indicate that RNAi is effective in reducing the levels of gliadins in wheat, which would be safer for gluten-intolerant consumers. However, it is still debatable if these wheat lines will become commercially viable or whether the discoveries will be converted into something of economic utility.

## 10. Applications of CRISPR/Cas9 and RNAi: A Comparative Analysis

**Figure 2:**
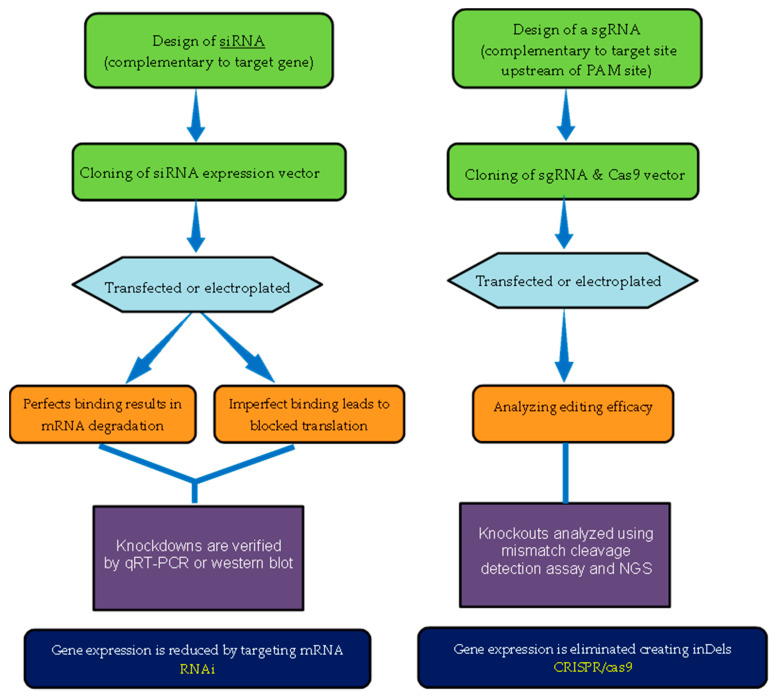
Schematic representation of RNAi and CRISPR/Cas9 experimental workflow. Abbreviations: NGS, next-generation sequencing; inDel, insertion–deletion mutation.

Gene modifications are powerful tools that have been widely used in past decades to understand fundamental biological processes of interest and their function. RNAi has previously been the major dominating genetic tool for manipulating genes and performing genetic function studies in various areas of crop development. However, the rapid growth and use of CRISPR/Cas9 have been successfully applied in many agronomic crops. Both RNAi and CRISPR/Cas9 are useful tools for modifying genomic DNA and changing genetic information, including gain-of-function and loss-of-function. CRISPR/Cas9 and RNAi are widely explored from a technical and methodological standpoint (Figure 2). A comparison of the scope of CRISPR/Cas9 and RNAi in research and practical studies is discussed below.

**Knockout vs. Knockdown**: CRISPR causes gene knockouts, which occur when DSB is made within the coding region of the gene [93]. This DSB triggers NHEJ or HDR [94]. RNAi reduces or knocks down gene expression at the post-transcriptional level by targeting RNA, where it generates a hypomorphic phenotype in contrast to the true null knockout that is possible with CRISPR/Cas9.
**Ease of Design**: The designing of a siRNA requires the sequence information of the corresponding mRNA transcript. siRNA is designed to target any transcript at almost any locus, but its activity is influenced by other factors such as the structure of the mRNA target region, base preferences, and overall siRNA G/C content. The design of a siRNA is a critical component of an effective RNAi experiment. CRISPR, on the other hand, requires information about the genomic DNA sequence. A CRISPR system such as CRISPR/Cas9 requires the protospacer adjacent motif (or PAM), a short DNA sequence required to cleave the targeted DNA. Depending on the type of Cas9, the PAM sequence recognizes the 5′-NGG-3′ site (where “N” can be any nucleotide base) [95].
**Timespan**: The mode of action differs between CRISPR/Cas9 and RNAi, which greatly impacts the duration of gene expression. siRNA knockdown exhibits significant gene repression within only 24 h of treatment. However, genome editing with CRISPR/Cas9 may result in a permanent effect, which usually requires the selection of cells with the desired InDels (insertion-deletion mutation) in all alleles, a time-consuming process depending on the specific need [96].
**Flexibility**: Targeted gene editing, especially CRISPR/Cas9, is heritable, i.e., once it introduces the change in the genome of the host cells, its physiological effect is passed on to the next generation. RNAi, unlike CRISPR/Cas9, does not result in a stable gene fragment, mutation, or inactive gene [97]. The in vivo application of RNAi is limited to instances where gene expression is suppressed post-transcriptionally.
**Off targets**: Since the discovery of RNAi, off-targets are one of its biggest limitations. siRNA induces the silencing of non-target mRNA with a limited sequence complementarity, via interaction with 3′UTR. However, it has been discovered that a single siRNA could potentially repress hundreds of transcripts with limited complementarity. However, the CRISPR/Cas9 system also has some sequence-specific target effects that can be overcome over a short period of time. This shortcoming was rectified through the use of the Cas9-nickase, a mutation in one of the Cas9 nucleases that reduces off targeting by 50-1500 fold [98]. While optimal siRNA design and chemical modifications have reduced the off-target activity of RNAi, a recent comparative study found that CRISPR/Cas9 is less susceptible to off-target effects than RNAi [99].

## 11. CRISPR/Cas9 Is a Method-of-Choice for Wheat Genome Editing

The recent emergence of multiple technologies for modifying gene structure has reformed agriculture and has resulted in improved that were not possible with conventional breeding procedures alone. These genetically modified crops have created huge economic and environmental benefits and are widely accepted across the world. Over the past decade, the RNAi technique has been widely used in both dicotyledon and monocotyledon to improve plant growth and productivity, impart resistance against pathogens, and create tolerance against various biotic stresses. RNAi or post-transcriptional gene silencing (PTGS) is a cellular mechanism conserved in most eukaryotic organisms that leads to the loss of functionality of a gene by blocking the messenger RNA (mRNA) molecules needed for protein formation.

Since RNA expression constructs are typically delivered as transgenes, through plant transformation, or as part of virus vectors, they must go through genetically modified organism (GMO) regulatory procedures to gain commercial approval. Several other techniques for stable genetic modifications, collectively known as gene-editing techniques, have been developed in parallel to the production of RNAi [100]. CRISPR is one such novel second-generation genome-editing system that has been exploited to generate desired mutations, facilitating the development of crops with any given desirable trait. In the last decade, due to its simplicity, speed, and efficiency, CRISPR/Cas9 has quickly become a standard technique for modifying endogenous genes in almost all crop species. The CRISPR/Cas9 system has target specificity, as the target sites are recognized by the Watson and Crick model, and the off-target sites are identified through sequence analysis [101]. CRISPR/Cas9 represents significant technical advances for genetic engineering, but attempts must be taken to increase its productivity in a variety of plant species with large, complex genomes.

While the utility of the CRISPR/Cas9 has been studied in many diploid plants, its applicability in polyploidy crops with complex genomes (wheat) is still a challenge. Wheat is an allohexaploid that consists of three sets of closely associated homogeneous genomes [37,59]. Therefore, simultaneously targeting three or even more copies of a gene is a problem for editing wheat genomes, and attempting to knock out any of a gene’s copies does not result in phenotypic modifications due to genome buffering. Wheat, on the other hand, which has a large genome and a high content of repetitive DNA (80–90%), makes it unusually recalcitrant to introduce targeted mutations. However, due to the availability of new orthologs of the Cas9 gene, sgRNA design in the CRISPR/Cas9 system can be effectively programmed to target several genes.

Another concern is that there are only a few wheat varieties that can be easily transformed, which restricts the use of CRISPR in wheat. However, there are well-established protocols for the transformation of the CRISPR/Cas9 construct using *Agrobacterium*-mediated and bombardment or biolistics delivery methods [35]. In addition, using recently designed CRISPR-based multiplex genome-editing toolkits, it is possible to accomplish simultaneous multiplex targeted modifications by co-transforming multiple sgRNAs. Evidence from published data shows that the CRISPR/Cas9 technique has been successfully applied to numerous wheat varieties to engineer novel agronomic traits associated with yield, quality, and resistance to biotic and abiotic stresses, etc. CRISPR/Cas9 is highly desirable for achieving the goal of editing α-gliadin genes in the development of wheat lines with fewer gluten genes and/or gluten genes with inactivated CD epitopes in bread wheat [13].

## 12. Discussion

Celiac disease is a complex disorder in which the function of a major non-genetic factor, i.e. ‘gluten’ has been well established. A life-long GFD is the sole cure for CD [7]. However, a GFD, on the other hand, is difficult to follow because gluten is a commonly used food additive that can be found in items that do not initially contain gluten [10,102]. Furthermore, gluten-free products can be less healthy nutritionally since they are made with high levels of fat and sugar to create a texture that resembles the normal and unusual viscoelastic properties of wheat. Additionally, studies have linked GFD to the lower consumption of dietary fiber, and some commercially available GFPs have lower vitamin B, folate, and iron content [103]. Moreover, the exclusion of gluten from the diet of CD patients reduces their QOL [11]. Rigorous efforts have been conducted to explore an alternative treatment that allows CD patients to consume wheat [17]. The use of a special wheat variety devoid of T-cell stimulatory epitopes may be a viable and successful alternative option. Currently, the only safe alternative would be the development of a “low-gluten/gluten-free” wheat variety that does not contain toxic peptides while retaining the basic properties of wheat [17,104,105].

Since bread wheat has a complicated hexaploid genome, the successful breeding of this crop is heavily reliant on the understanding of functional genomics. Advanced crop functional genomics, which can show how wheat genetics determine function, must now be complemented with existing modern breeding efforts. Plant biologists, based on their understanding of functional genomics, can alter the structures and functions of selected key genes through “genetic manipulation” based on their understanding of functional genomics. RNAi and CRISPR/Cas9 are two advanced technologies that can be used to modify or remove CD inducing epitopes from wheat gluten. The RNA silencing technique shows favorable results in this regard. Several research groups have explored the possibilities of using RNAi in silencing the toxic fragments of gliadin and have found promising results [84,90,106]. In a fundamental study, Gil-Humanes et al. used RNA interference to reduce gliadin gene expression by 97%, therefore preventing the stimulation of T cells from CD patients without compromising seed germination or dough quality [90]. The RNAi wheat line (E82) developed by Javier Gil-Humanes and colleagues was exceptional because of its low ability to produce an immunogenic response and its ability to retain its organoleptic and agricultural properties. The study was conducted in volunteer NCGS patients and was compared with a GFD to test the acceptability, digestibility, and safety of the bread made from the wheat flour of the E82 line with all of the gliadins being strongly downregulated. Furthermore, in non-celiac wheat sensitivity patients, eating bread made with this low-gliadin line encourages a stronger gut microbiota profile than gluten-free bread [91].

Since the transgenic RNAi construct persists in the wheat genome to silence the genes, these plants are subjected to GM control, which is costly, time-consuming, and unpredictable in the European Union (EU) [21,26,107]. Unlike other breeding methods, the implementation of genetic transformation is strongly regulated in the EU. This contradicts the fact that the cultivation of GMOs is essentially prohibited in the EU, but importation is permitted [108]. As a result of this stringent regulation, the general population is concerned about GMOs on a variety of levels, including their environmental impact and whether GM foods pose any health risks.

Emerging targeted genome-editing technologies offer plant breeders a new and effective tool. In terms of genome editing, SSNs have been used to alter the target position of genes present in the genome. SSN, similar to CRISPR/Cas9, causes DSB, which can be repaired using an NHEJ or HR [54,109]. Unlike transgenic modifications, which require the insertion of foreign DNA sequences into a genome, gene editing may produce genetic variation through precise and direct changes in the genes of interest without integrating foreign DNAs or, if so, null segregants containing no recombinant DNA but that maintain the desired mutations and that can be easily retrieved. Instead of being categorized as GMOs, such edited plants could be considered non-transgenic plants. Moreover, it is expected that the Court of Justice of the European Union (ECJ) will exempt CRISPR/cas9 modified crops from the existing European law that has limited the planting and sale of GM crops [110].

In plants, CRISPR/Cas9 has already been shown to be a very highly efficient genome-editing system [111,112,113]. The hexaploid genome and large genome size are the major obstacles to CRISPR use in wheat biology. However, because of the high efficiency of CRISPR/Cas9 it is possible to acquire mutations in multiple genomes in a single polyploid plant. Finally, multiplexed genome editing using the CRISPR/Cas9 library can be easily accomplished using the monomeric Cas9 protein and a variety of sequence-specific gRNAs [25,59]. Moreover, genome editing through CRISPR/Cas9 entails a few simple steps that enable smaller laboratories with basic plant transformation abilities to perform genome editing in crop plants. The ease of use of CRISPR/Cas9 programming and its potential for multiplexed target identification have fueled the success of this low-cost and easy-to-use technology. According to some research, while CRISPR/Cas9 can cleave a target site, it can also cleave sites that do not match the target site [61]. In gene therapy, this off-target effect is a major problem, but it may not be a concern in plant biotechnology. Back-crossing or crossing with wild-type plants could be used to remove the putative off-target mutations. Furthermore, the use of web-based software to develop target sites is advised in order to mitigate off-target mutations by exploiting computation.

Susana Sánchez-León et al. utilized CRISPR/Cas9 genome editing technology to reduce the number of α-gliadins in the seed kernel precisely and effectively, resulting in gluten-free bread and durum wheat lines [12]. Interestingly, the bread wheat line (plant 10) had the highest decline in α-gliadins (82%) and γ-gliadins (92%) as well as the highest overall gliadin reduction (82%). Amongst the durum wheat lines, plant 2 had the highest overall gliadin reduction (69%). By improvising the current intricacies in the methodology, it is possible to develop a safe variety of wheat for CD patients. If such gluten-free wheat maintains its natural taste, it would be easier for CD patients to adhere a completely to the GFD. About 50% of CD patients do not follow a strict GFD for multiple reasons, including the unavailability of gluten-free food and the appalling palatability of the GFD, etc. [8,114]. A safe wheat variety for CD patients would be helpful to eradicate this problem, and CRISPR/Cas9 technology has the potential to produce such a variety of wheat [26]. However, CRISPR-modified wheat flour may lead to problems such as dough formation that need to be resolved. Nonetheless, multiple studies support the fact that CRISPR/Cas9-mediated gene editing has overcome the current wheat genome complexity for genetic improvement (Table 1). The use of CRISPR/Cas9 for gene knockout and the Cas9 system for the expression regulation of any gene of interest would aid in the development of non-transgenic wheat plants. CRISPR technology is evolving, and existing systems are being engineered to include innovative capabilities. Moreover, exciting new CRISPR systems with novel functions are also being discovered.

## 13. Conclusions

The CRISPR/Cas9 gene-editing system is capable of editing the complex hexaploid wheat genome (*T. aestivum*). The availability of whole-genome sequence information for wheat along with the advancements in the CRISPR/Cas9 technique could provide possibilities for the development of a “hypo-immunogenic-wheat variety”. CRISPR/Ca9 could be a breakthrough for providing a promising dietary treatment for celiac disease. However, until now, only a limited number of studies have applied the CRISPR/Cas9 system to develop low-gluten wheat. Further studies are required to apply the CRISPR/Cas9 gene-editing system efficiently for the development of a celiac-safe wheat variety and to establish it as a “tool to celiac safe wheat”.

## Figures and Tables

**Table 1:** Summary of functionally validated CRISPR/Cas9-based genome editing in wheat varieties.

S. No	Cultivar or Genotype	Target Gene (s)	Gene Function	Delivery Mode	SgRNA Promoter Used	Reference
1	*T. aestivum* cv. Cadenza	*TaASN2*	Genes encode for asparagine synthetase enzyme required in asparagine synthesis	Biolistic transformation	*Ubi-1*	Raffan et al., (2021) [70]
2	*T. aestivum* line H29 cv. Fielder & Ningchun4	*TaWaxy* & *TaMTL*	Pollen-specific phospholipase	*Agrobactrium tumefaciens* mediated transformation	*OsU6a*, *TaU3*, and *TaU6*	Liu et al., (2020) [71]
3	Wheat variety CB037	*TaNP-A1, TaNP-B, TaNP-D1*	Expression in the tapetum and required for male fertility	Biolistic and protoplast mediated transformation	*TaU6* and *TaU3*	Li et al., (2020) [72]
4	Common wheat (*T. aestivum* L.)	*TaQsd1, TraesCS4A02G110300 (IWGSC 2018)*	Control seed dormancy in wheat	Biolistic transient expression and *A. tumefaciens* mediated transformation	*TaU6*	Kamiya et al., (2020) [69]
		*TaLOX2*	Encodes for lipoxygenase 2; grain development and growth			
5	*T. aestivum* cv. Fielder	*TaABCC6* & *TaNFXL1*	Susceptibility to Fusarium head blight (FHB)	Protoplast transformation	*TaU6*	Cui et al., (2019) [73]
		*TansLTP9.4*	FHB resistance			
6	*T. aestivum* cv. Fielder	*EPSPS*	The key enzyme involved in the metabolism of aromatic amino acid through the shikimate pathway	Protoplast transformation	*TaU6*	Arndell et al., (2019) [74]
7	*T. aestivum* cv. Fielder	*TaPinb*	Control grain hardness	*A. tumefaciens* (EHA105) mediated transformation	*TaU3*	Zhang et al., (2019) [67]
		*TaDA1*, *TaDA2*	Negative regulates seed and organ size			
		*TaNCED1*	Key enzyme in ABA biosynthesis pathway that confers resistance to drought stress			
8	*T. aestivum* cv. Fielder	*TaQsd1*	Control seed dormancy in wheat	*A. tumefaciens* (EHA101) mediated transformation	*OsU6*	Abe et al., (2019) [68]
9	*T. aestivum* cv. Kenong199 or Kenong9204	*TaALS*, *TaACCase*	The absence of the gene provides herbicide tolerance	Biolistic transformation	*TaU6*	Zhang et al., (2019) [75]
10	*T. aestivum* cv. Fielder & cv. Gladius	*TaMs1*	Encodes a GPI, which is required for pollen exine development	*A. tumefaciens* mediated transformation	*TaU6*	Okada et al., (2019) [65]
11	*T. aestivum* cv. Fielder	*TaCKX2*-*1*, *TaGLW7*, *TaGW2*, *TaGW8*	Wheat grain-regulatory genes	*A. tumefaciens* mediated transformation	*TaU6*	Zhang et al., (2019) [76]
12	*T. aestivum* cv. Fielder	*TaPin a* & *b*	Control grain hardness and contributes to anti-fungal properties	*A. tumefaciens* mediated transformation	*TaU6* & *TaU3*	Zhang et al., (2018) [44]
		*TaWAXY* or GBSS	Key enzyme in amylase biosynthesis			
		*TaDA1*	Negatively regulates seed and organ size by restricting the period of cell proliferation			
13	*T. aestivum* cv. Bobwhite	*TaGW2*	Negative regulator of grain weight, grain size enlargement, especially increased kernel width	Protoplast transformation	*TaU6*	Wang et al., (2018) [59]
		*TaLpx*-*1*	Encodes 9-lipoxygenase, silencing results in resistance to *Fusarium graminearum*			
		*TaMLO*	Knockout mutants provide resistance to powdery mildew			
14	*T. aestivum* cv. Chinese Spring	*TaPDS*	Reduction or loss of function results in a photobleaching phenotype	*A. tumefaciens* mediated transformation	*TaU6*	Howells et al., (2018) [66]
15	*T. aestivum* cv. Fielder or SBC0456D	*TaMs45*	Contribute to male fertility	*A. tumefaciens* mediated transformation	*TaU6*	Singh et al., (2018) [77]
16	Bread wheat, BW208 & THA53, & Durum wheat cv. Don Pedro	α-gliadin	Storage protein, adds to dough viscosity/plasticity and contains immunogenic epitopes for CD	Biolistic transformation	*TaU6*	Sánchez-León et al. (2018) [12]
17	*T. aestivum* cv. Chinese spring	*TaDREB2*	TF induced under water-deficient condition	Protoplast transformation	*TaU6*	Kim et al., (2018) [60]
		*TaERF3*	TF promotes tolerance under salt and drought stress			
18	*T. aestivum* cv. Bobwhite & AC Nanda	*TaLox2*	Encodes for lipoxygenase enzyme, which hydrolyzes linoleic acid, α-linolenic acid, and arachidonic acid	Neon transfection of protoplasts and microspores	*TaU6*	Bhowmik et al., (2018) [78]
19	*T. aestivum* cv. Bobwhite	*TaUbi, TaMLO*	Majorly responsible for powdery mildew vulnerability	WDV and Biolistic transformation	*TaU6*	Gil-Humanes et al., (2017) [63]
20	*T. aestivum* cv. Kenong 199	*TaGW2*-A1, -B1 & -D1	Negatively regulates grain weight and width	Biolistic transformation	*TaU6*	Liang et al., (2017) [31]
21	*T. aestivum* cv. Bobwhite & cv. Kenong199	*TaGASR7*	Gene controls the expression of grain length with pleiotropic effects on grain weight and yield	Biolistic transformation	*TaU6*	Zhang et al., (2016) [79]
		*TaDEP1*	Gene expression controls panicle size			
		*TaLOX2*	Encodes for lipoxygenase 2 and plays a critical role in grain storage and seed vigor			
		*TaNAC2*	TF promotes multiple abiotic stresses tolerance			
		*TaPIN*	Encodes for puroindoline gene and plays an important role in controlling the grain hardness			
		*TaGW2*	Negative regulator of grain weight, grain size enlargement, and especially increased kernel width			
22	*T. aestivum* L.	*TaMLO-A1, TaMLO-B1* & *TaMLO-D1*	Loss of function confers resistance to Powdery mildew	Biolistic transformation	*TaU6*	Wang et al., (2014) [62]
23	*T. aestivum*	*TaINOX*	Biogenesis of plant cell wall	*A. tumefaciens* (GV3101) mediated transformation	TaU6 and CaMV35s	Upadhyay et al., (2013) [80]
		*TaPDS*	Involved in carotenoid biosynthesis that protects chlorophyll from photobleaching			

ABA, abscisic acid; TF, transcription factor; CD, celiac disease; EPSPS, 5-enolpyruvylshikimate-3-phosphate synthase; GPI, glycosylphosphatidylinositol; *OsU*, *O. sativa* small nucleolar RNA (snoRNA) promoters; *TaU*, *T. aestivum* snoRNA promoters; *Ubi-1*, *Z. mays* ubiquitin promoter.

## Data Availability

Not applicable.
